# Orientation, elastic interaction and magnetic response of asymmetric colloids in a nematic liquid crystal

**DOI:** 10.1038/s41598-018-36467-0

**Published:** 2019-01-14

**Authors:** Dinesh Kumar Sahu, Thriveni G. Anjali, Madivala G. Basavaraj, Jure Aplinc, Simon Čopar, Surajit Dhara

**Affiliations:** 1 School of Physics, University of Hyderabad, Hyderabad, 500046 India; 2 Department of Chemical Engineering, Indian Institute of Technology Madras, Chennai, 600 036 India; 3 Faculty of Mathematics and Physics, University of Ljubljana, Jadranska 19, 1000 Ljubljana, Slovenia

## Abstract

Colloidal particles in nematic liquid crystals create elastic distortion and experience long-range forces. The symmetry of elastic distortion and consequently the complexity of interaction strongly depends largely on the liquid crystal anchoring, topology and shape of the particles. Here, we introduce a new nematic colloidal system made of peanut-shaped hematite particles. We report experimental studies on spontaneous orientation, mutual interaction, laser assisted self-assembly and the effect of external magnetic fields on the colloids. Majority of the colloids spontaneously orient either parallel or perpendicular to the nematic director. The colloids that are oriented perpendicularly exhibit two types of textures due to the out of plane tilting, which is corroborated by the Landau-de Gennes Q-tensor modelling. The transverse magnetic moment of the peanut-shaped colloids is estimated by using a simple analysis based on the competing effects of magnetic and elastic torques.

## Introduction

Colloidal particles dispersed in anisotropic solvents such as liquid crystals (LCs) are a fascinating class of soft matter. There has been a surge of activity in this field ranging from fundamental to applied issues^1–6^. The dispersed particles experience elastic forces which are very different than their counterparts in isotropic fluids. The forces are long-range, anisotropic and depend on the symmetry of the elastic distortion created by the particles. The competition between the uniform director field (average direction of molecular orientation) and anchoring on the surface leads to the induction of topological defects in the vicinity of the particles^7–13^. In case of spherical colloids the defects appear as points or rings depending on the type and strength of surface anchoring and confinement^13,14^. The colloids together with defects are known as elastic dipoles or quadrupoles, based on the similarity of the director fields with their electric counterparts^4^. In addition, other kinds of defects such as boojums^15,16^ and vortex^17,18^ are also observed in different anchoring and confinement conditions. Based on the interplay of elastic interaction and topological defects a variety of self-assembled structures such as chains^3^, two and three dimensional crystals^6,8^, entangled clusters^9,19^, knotted and linked colloids^19–22^ are reported. It has been shown that the symmetry of the elastic interaction and the resulting colloidal assembly can be markedly different due to the the shape of the colloids. In fact there are several reports on the induced defect and complex interaction of nonspherical microparticles such as rod-shaped^11,23^, star-shaped^24^, rectangular^25,26^, triangular^27^, square, bullet^28^ and doughnut-shaped^27^ in nematic crystals.

The self-assembly of magnetic colloids that can be controlled or manipulated by external magnetic field is very promising and comparatively less explored than nonmagnetic colloids. Brochard and de Gennes first theoretically studied the magnetic particles in nematics and proposed the idea of a ferronematic^29^. Lately there are several experimental studies on magnetic microparticles^30,31^, nanowires^32,33^ and nanoparticles in nematic liquid crystals^34–36^. However, after the discovery of true ferronematic by Mertelj *et al*.^37^, it has created immense interests^38–40^. Here we introduce a new nematic colloidal system –peanut-shaped hematite colloids in nematic liquid crystals. The assembly and interactions of these particles have been studied in aqueous medium. They interact via dipolar interaction and also experience a magnetic torque under the influence of external magnetic field. In isotropic solvents like water they exhibit complex self-assembled and ordered structures^41,42^. It is expected that in anisotropic solvent such as in nematic liquid crystals we can engender the diversity of the interaction, orientation and hence the self-assembly of the peanut-shaped colloids by exploiting the combined effect of magnetic and elastic interactions. The elastic interaction is a consequence of elastic distortion that is primarily governed by its shape. A peanut shape benefits from anisotropic interactions, while retaining smooth sphere-like geometry on both ends, which helps in formation of regular chains and lattices. With this aim we study spontaneous orientation, induced defects, anisotropic elastic interactions, directed self-assembly and magnetically driven rearrangement of peanut-shaped magnetic colloids. The microscopic textures due to the elastic distortion are simulated by the Landau-de Gennes Q-tensor modelling. By balancing the liquid crystal imposed forces with applied magnetic field we determine the elastic torque which leads to the quantitative measurement of the magnetic dipole moment of the peanut-shaped particles.

## Results and Discussion

**Figure 1:**
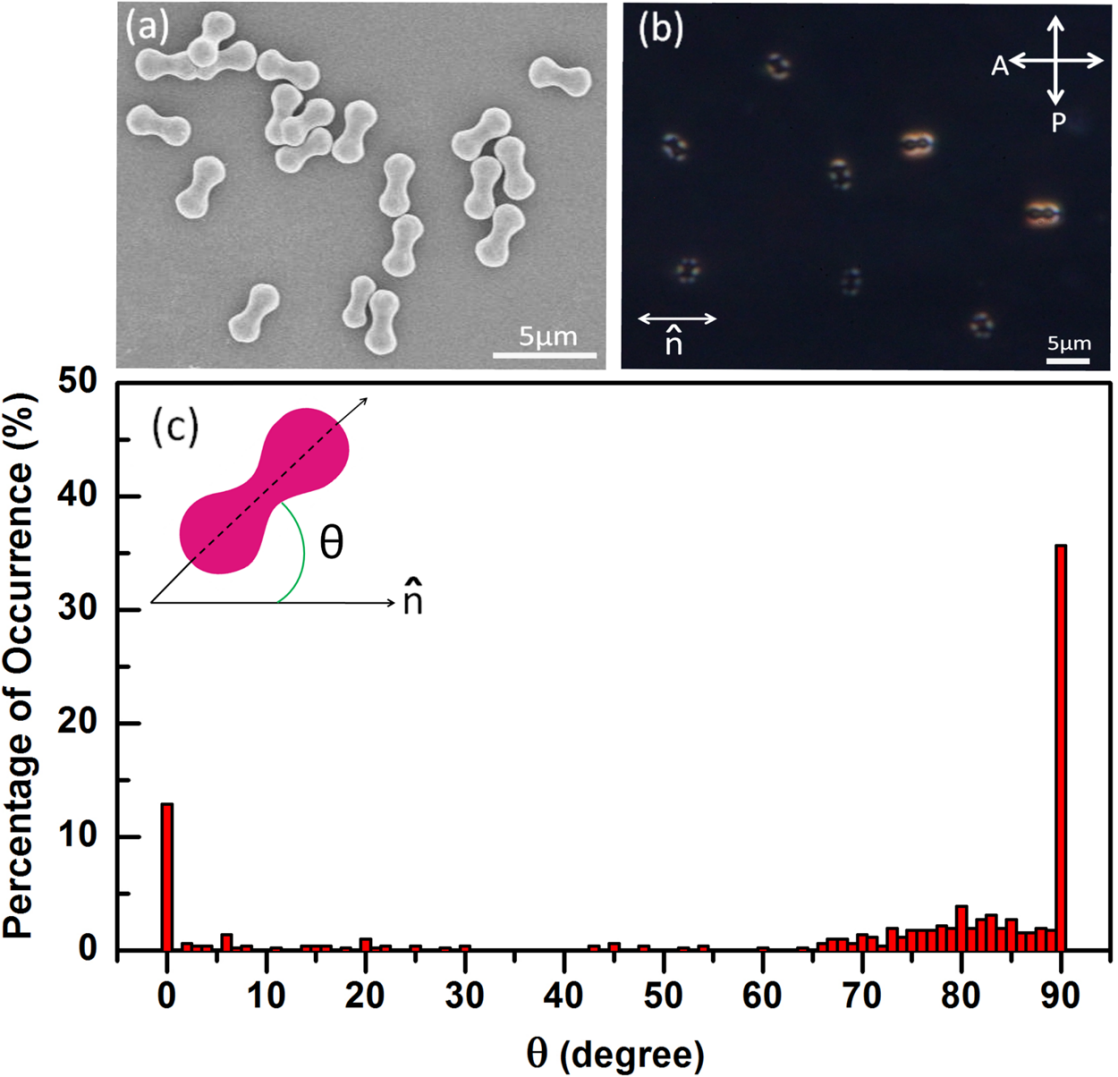
(**a**) SEM micrograph of peanut-shaped particles spreaded on a clean glass plate. (**b**) POM texture of dispersed particles in 5CB, where double headed arrow above $\hat{n}$ indicates the nematic director and crossed double headed arrows indicate orientation of polariser and analyser. (**c**) The percentage of number of colloids oriented at different angles with respect to the director ($\hat{n}$). About 250 particles are analysed. Cell thickness *d* = 9.5 μm.

The particles are first characterised by scanning electron microscope (SEM). Fig. 1(a) shows a SEM micrograph of the peanut-shaped hematite particles. The average length and the lobe diameter of these particles are about 3.2 μm and 1.54 μm respectively. The mixture of peanut-shaped colloids and 5CB is introduced in planar cells. Figure 1(b) shows a texture with a few dispersed colloids taken by polarising optical microscope (POM). It is observed that the colloids orient themselves with their long axis at all possible angles with respect to the nematic director, $\hat{n}$. The statistical analysis of orientation of about 250 particles is shown in Fig. 1(c). About 35% colloids are oriented perpendicular to the director and 13% are oriented parallel to the director. The remaining colloids are oriented at all other angles (0^o^ < *θ* < 90°). This possibly indicates a slight differences in anchoring strength of the particles.

**Figure 2:**
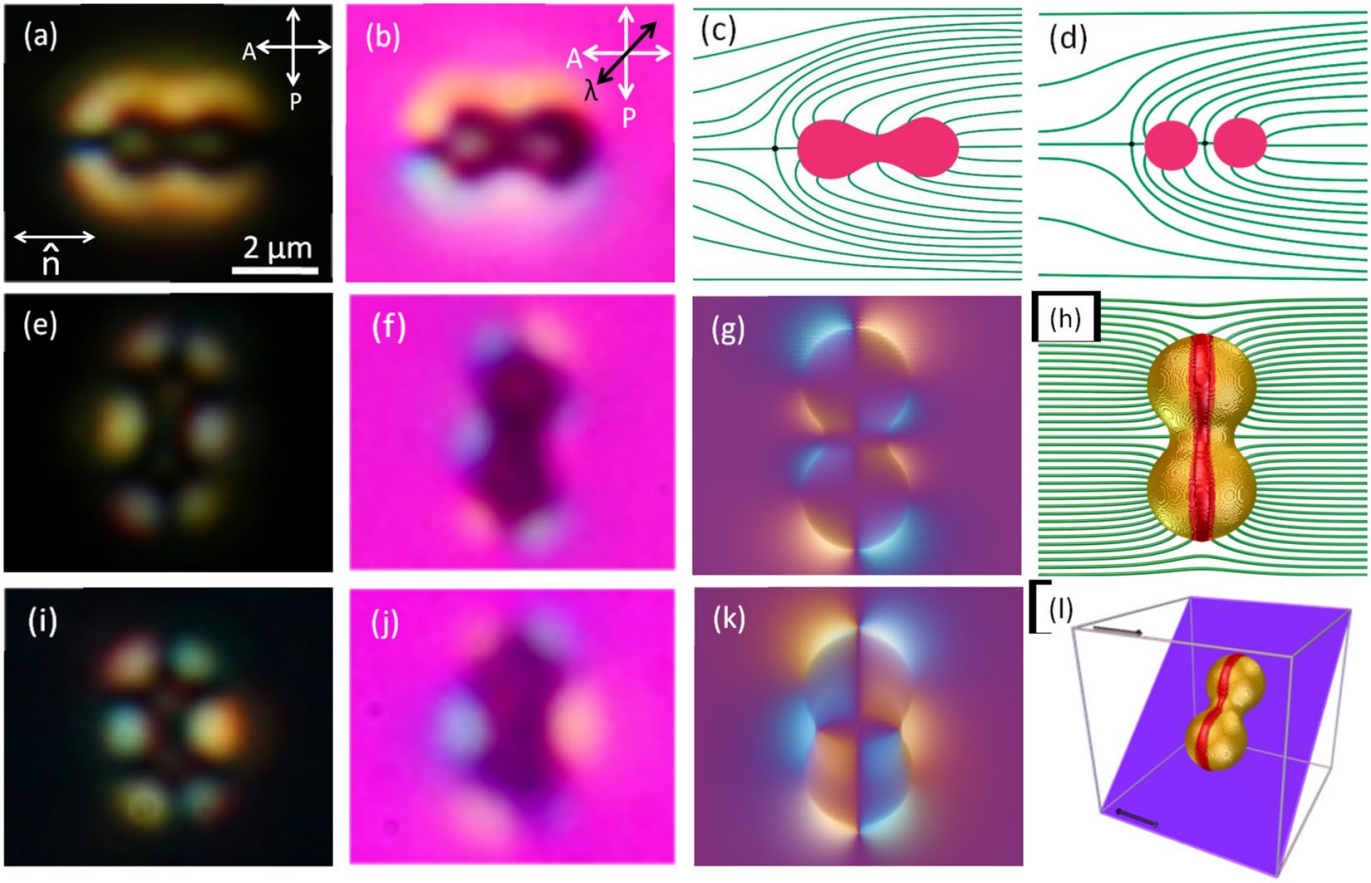
(**a**,**e**,**i**) POM images of particles oriented parallel and perpendicular to the director. (**b**,**f**,**j**) Corresponding images obtained by keeping the fast axis of the *λ*-plate (530 nm) at 45° with respect to the polariser. (**c**) Schematic diagram of elastic dipolar-type deformation around a colloid with parallel orientation. (**d**) Schematic diagram of elastic deformation around a pair of spherical dipolar colloids for comparison. Perpendicular colloids with (**e**) nontilted and (**i**) tilted orientations. Simulated POM images with *λ*-plate of perpendicular colloids with (**g**) nontilted and (**k**) tilted orientations. (**h**) Director image from the simulation showing elastic distortion and ring defect around the nontilted colloid (thick-red band along the length). (**l**) Schematic diagram of a tilted colloid in 3D perspective. In this configuration, the particle is positioned near the bottom of the cell. The black arrows near the edges of the box indicate the rubbing direction.

The elastic distortion of a few isolated colloids which are either parallel or perpendicular to the director are investigated with a 60× objective and numerical aperture (NA) of 1.1. To get qualitative information about the anchoring and the induced defects, a retarder wave plate (530 nm) was introduced into the optical path between the polariser and the sample stage making the fast axis 45° with respect to the director and the corresponding image is shown in Fig. 2(b). The molecules, parallel or perpendicular to the slow axis of the wave plate, transmit blue or yellow colour representing the clockwise or anticlockwise orientation with respect to the director, respectively.

Figure 2(a) shows the POM micrograph of a particle oriented parallel to the director. It induces a hyperbolic hedgehog point defect and elastic distortion similar to that of spherical micro-particles, excepting the presence of another point defect between them (see Fig. 2(d)). The point defect is slightly extended, possibly due to the shape or the aspect ratio of the particle. A schematic diagram of the director field around the particle is shown in Fig. 2(c).

**Figure 3:**
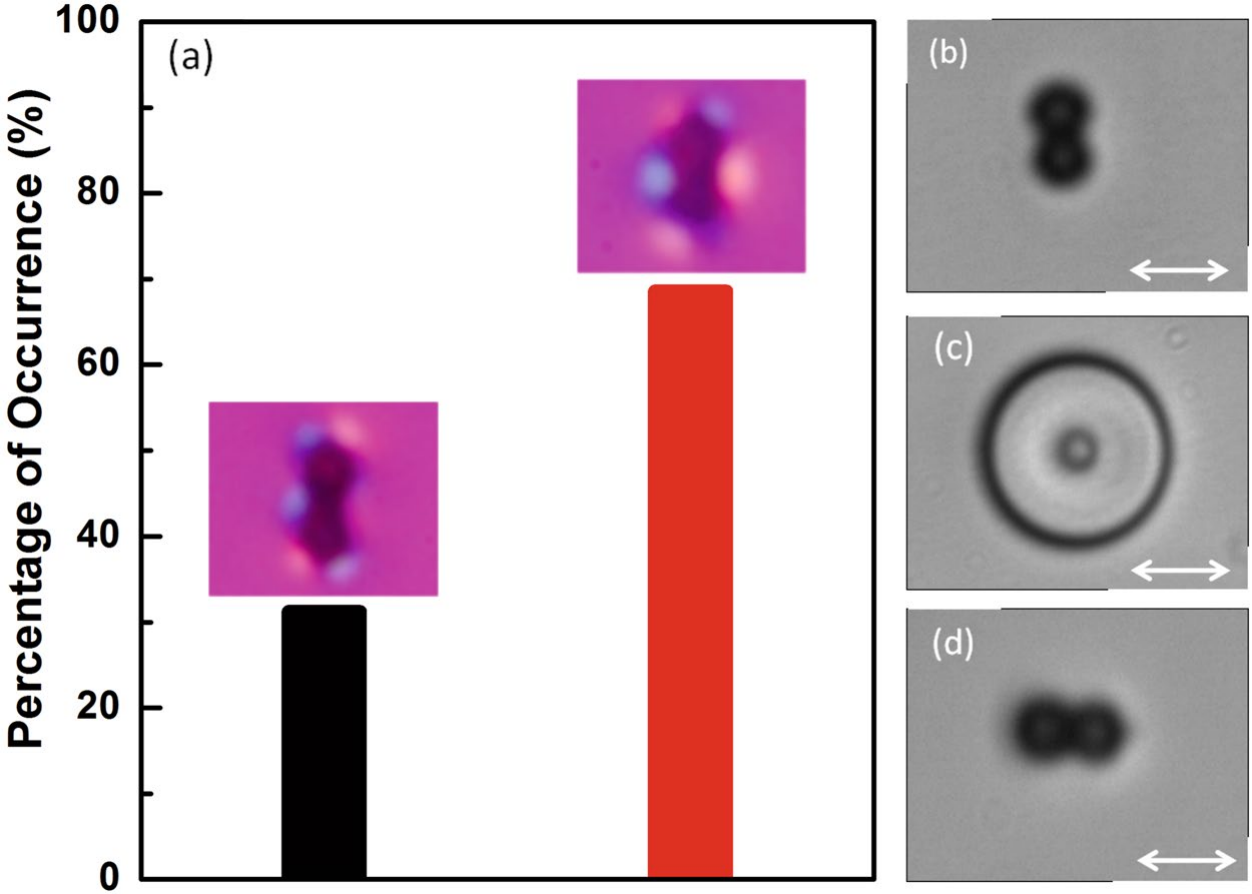
(**a**) Statistical analysis of perpendicular colloids on the basis of *λ*-plate images. Black and red bars represent percentage of nontilted and tilted colloids. (**b**–**d**) Sequence of CCD images showing that the orientation of a particle is changed by using a laser tweezer from vertical to horizontal.

POM micrographs of two particles oriented perpendicular to the director are shown in Fig. 2(e,i). Unlike the colloids that are oriented parallel to the director, the defects in this case are not easily identifiable. They exhibit two different retardation profiles when observed under *λ*-plate. In type-I, a typical sequence of blue and yellow colours that surrounds the particles (similar to that is observed in case of spherical colloids) suggests homeotropic anchoring of the liquid crystal molecules (Fig. 2(f)). Figure 2(g) shows the corresponding simulated image, where the particle is surrounded by a −1/2 disclination ring (Saturn ring) around its larger perimeter. The surrounding elastic distortion resembles to that of a pair of quadrupolar colloids except the concave surface around the neck. The *λ*-plate image of a type-II colloid shown in Fig. 2(j) is noticably different than that of type-I (Fig. 2(f)) though their POM images look similar (Fig. 2(e,i)). Careful observation shows that not all the particles that are oriented perpendicular to the director appear to have same length while dispersed in liquid crystals (Fig. 1(b)). The type-II colloids appear somewhat smaller than that of type-I, suggesting that the type-II colloids are tilted with respect to the plane of the cell. Based on this idea we carried out computer simulation for different tilt angles and vertical particle positions. Figure 2(k) shows the corresponding simulated *λ*-plate image of the tilted particle associated with the Saturn ring defect where the upper part of the particle is tilted 45^o^ out of the plane of paper and the lower part is set close to the bottom glass plate. In this configuration, the blue and yellow colour coincides best with that of the experiment (Fig. 2(f)). This suggests that the type-II relies on the interaction with the cell walls for stability, while the type-I is floating in the bulk. We have also done a statistical analysis of the two types of particle configurations. Fig. 3(a) shows that among the particles that are oriented perpendicular to the director nearly 70% are type-II i.e., tilted and remaining 30% are parallel to the glass plate. However, the orientation of the particles can be changed from parallel to perpendicular or from type-I to type-II or vice versa by using the optical tweezer as shown in Fig. 3(b–d).

**Figure 4:**
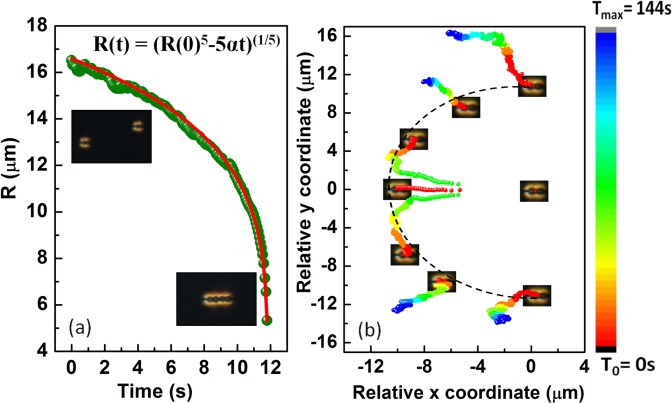
(**a**) The variation of center to center separation distance between two peanut-shaped particles with parallel dipolar configuration as a function of time. Red line is the best fit to the equation: $R(t)={(R{\mathrm{(0)}}^{5}-5\alpha t)}^{\mathrm{1/5}}$ with *α* = 2.1 × 10^4^ μm^5^/s. (**b**) Time coded trajectories of a dipolar particle from different angles with respect to another at the centre. Each trajectory is recorded for time interval from *T*_0_ = 0 s to *T*_*max*_ = 144 s.

**Figure 5:**
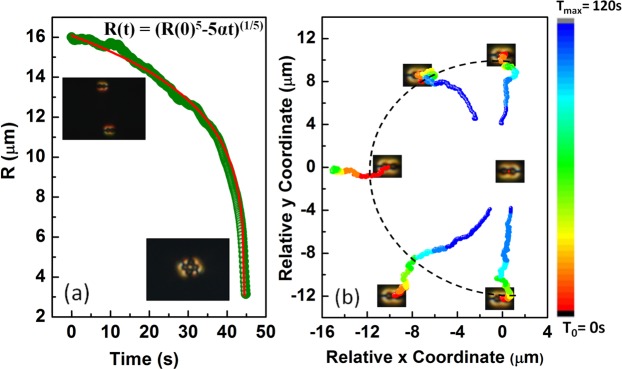
(**a**) Variation of equilibrium separation with time between two peanut-shaped particles with antiparallel elastic dipolar configurations. Red line is the best fit to the equation: $R(t)={(R{\mathrm{(0)}}^{5}-5\alpha t)}^{\mathrm{1/5}}$ with *α* = 4.8 × 10^3^ μm^5^/s. (**b**) Time coded trajectories of a dipolar particle from different angles with respect to another at the centre. Each trajectory is recorded for time interval from *T*_0_ = 0 s to *T*_*max*_ = 120 s.

The elastic interaction between two particles is studied using a video microscopy technique. Two dipolar colloids are brought a few micrometers apart with the help of the optical tweezer and left free to evolve. Figure 4(a) shows the variation of separation *R*(*t*) between two colloids with time. In this case, the attractive elastic force (*F*_*e*_) is balanced by the viscous drag force acting opposite to *F*_*e*_, hence *F*_*e*_ + *F*_*drag*_ = 0, where *F*_*drag*_ = −*ζdR*(*t*)/*dt*, ζ being the drag coefficient. The interaction force between two dipolar colloids is given by *F*_*e*_ = −*k*/*R*^4^, where *k* is a constant and depends on the mean elastic constant of the medium, size and shape of the particles. The time dependent inter-particle separation is given by $R(t)={(R{\mathrm{(0)}}^{5}-5\alpha t)}^{\mathrm{1/5}}$, where *α* = *K*/*ζ* and *R*(0) is the initial separation at *t* = 0 s^43^. The red line in Fig. 4(a) shows the best fit to the experimental data. Figure 4(b) shows time coded trajectories of a particle when it approaches another particle from different angles with parallel orientation of their dipolar axes. They have attractive interaction within about ±30° angle along the joining line and repulsive at other angles. When the dipolar particles approach along the broad-side on position with opposite orientations (Fig. 5(a)), they exhibit repulsive interaction within ±45° with respect to the joining line and attractive at other angles (see Fig. 5(b)). The overall anisotropic interaction of two peanut-shaped colloids are almost similar to that of spherical colloids except the numerical values of the interaction time and the range.

**Figure 6:**
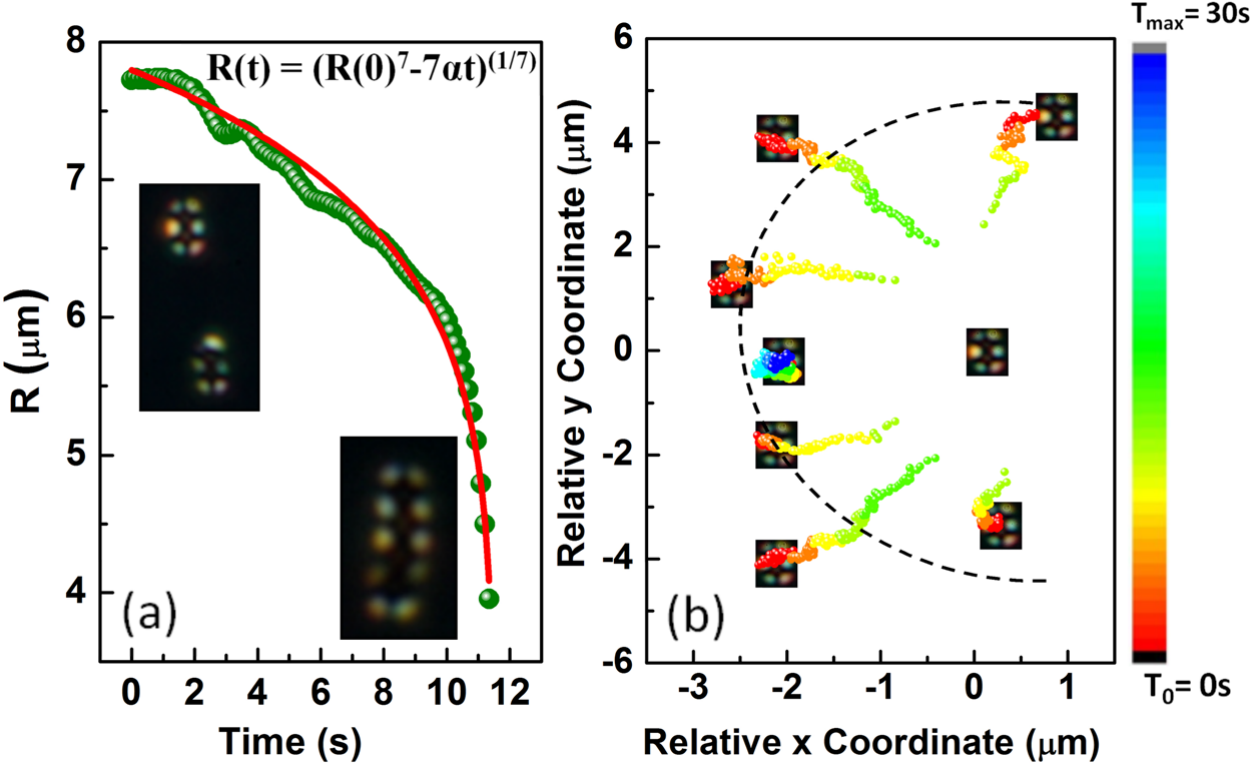
(**a**) Variation of equilibrium separation between two peanut-shaped particles with tilted elastic quadrupolar configurations. Red line is the best fit to the equation: $R(t)={(R{\mathrm{(0)}}^{7}-7\alpha t)}^{\mathrm{1/7}}$ with *α* = 2.1 × 10^4^ μm^7^/s. (**b**) Time coded trajectories of a tilted quadrupolar particle from different angles with respect to another at the centre. Each trajectory is recorded for time interval from *T*_0_ = 0 s to *T*_*max*_ = 30 s.

**Figure 7:**
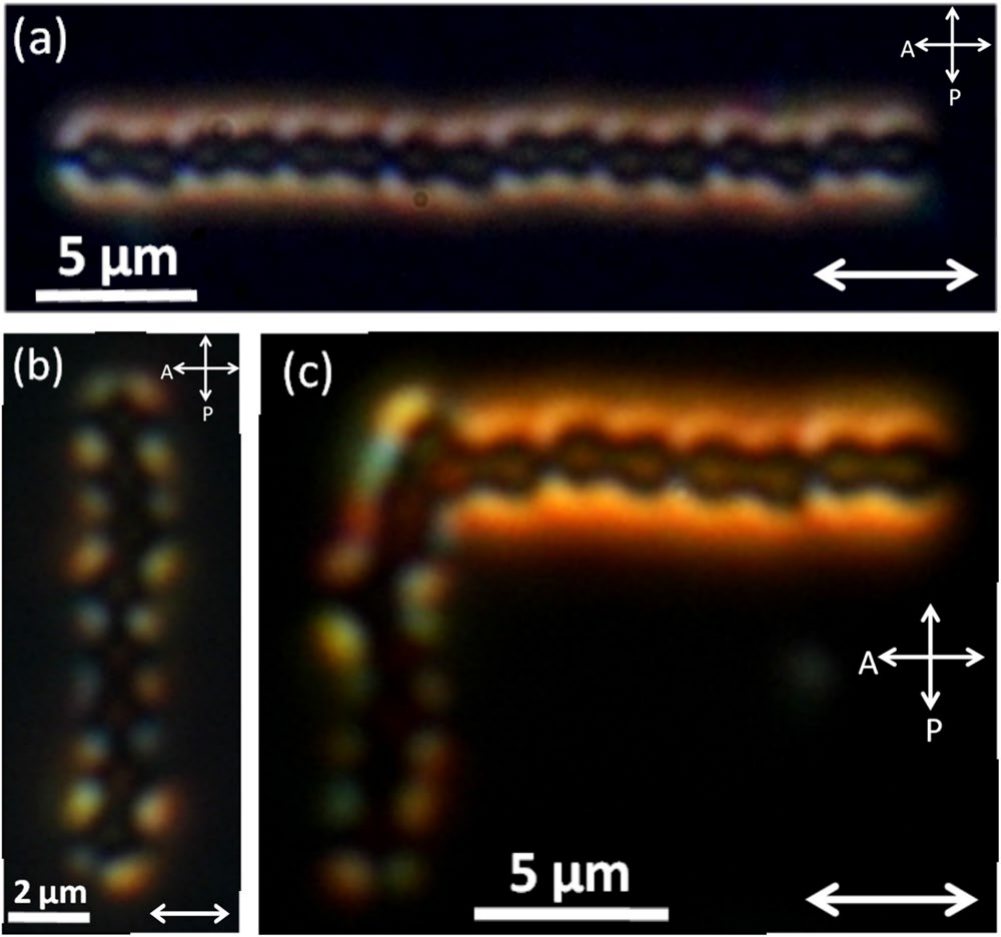
(**a**) Laser tweezer assisted self-assembly of dipolar and quadrupolar peanut-shaped colloids. (**b**) A linear chain of dipolar colloids parallel to the director. (**b**) A linear chain of tilted quadrupolar colloids normal to director. (**c**) L-shaped structure obtained by combining the two chains.

Pairs of perpendicular colloids (both types) show quadrupolar interaction (i.e *F*_*e*_ = −*k*/*R*^6^). Figure 6(a) shows the variation of inter-particle separation with the best fit to the corresponding equation, $R(t)={(R{\mathrm{(0)}}^{7}-7\alpha t)}^{\mathrm{1/7}}$. Figure 6(b) shows that except along the joining line of two colloids, they have attractive but short-range interaction. We prepared linear chains with a few colloids using the optical tweezer. Figure 7(a,b) shows self-assembled chains of peanut-shaped colloids with dipolar and quadrupolar defect configurations. The dipolar chain is oriented parallel to the director and the quadrupolar chain is oriented perpendicular to the director. Two orthogonal chains are guided by the laser tweezer to combine and form a L-shaped structure (Fig. 7(c)). This can be used as a building block for making complex colloidal self-assembled structures, which are responsive to external magnetic field. It may be mentioned that the orthogonal orientation of colloidal chains of spherical particles are not stable in nematic liquid crystals.

**Figure 8:**
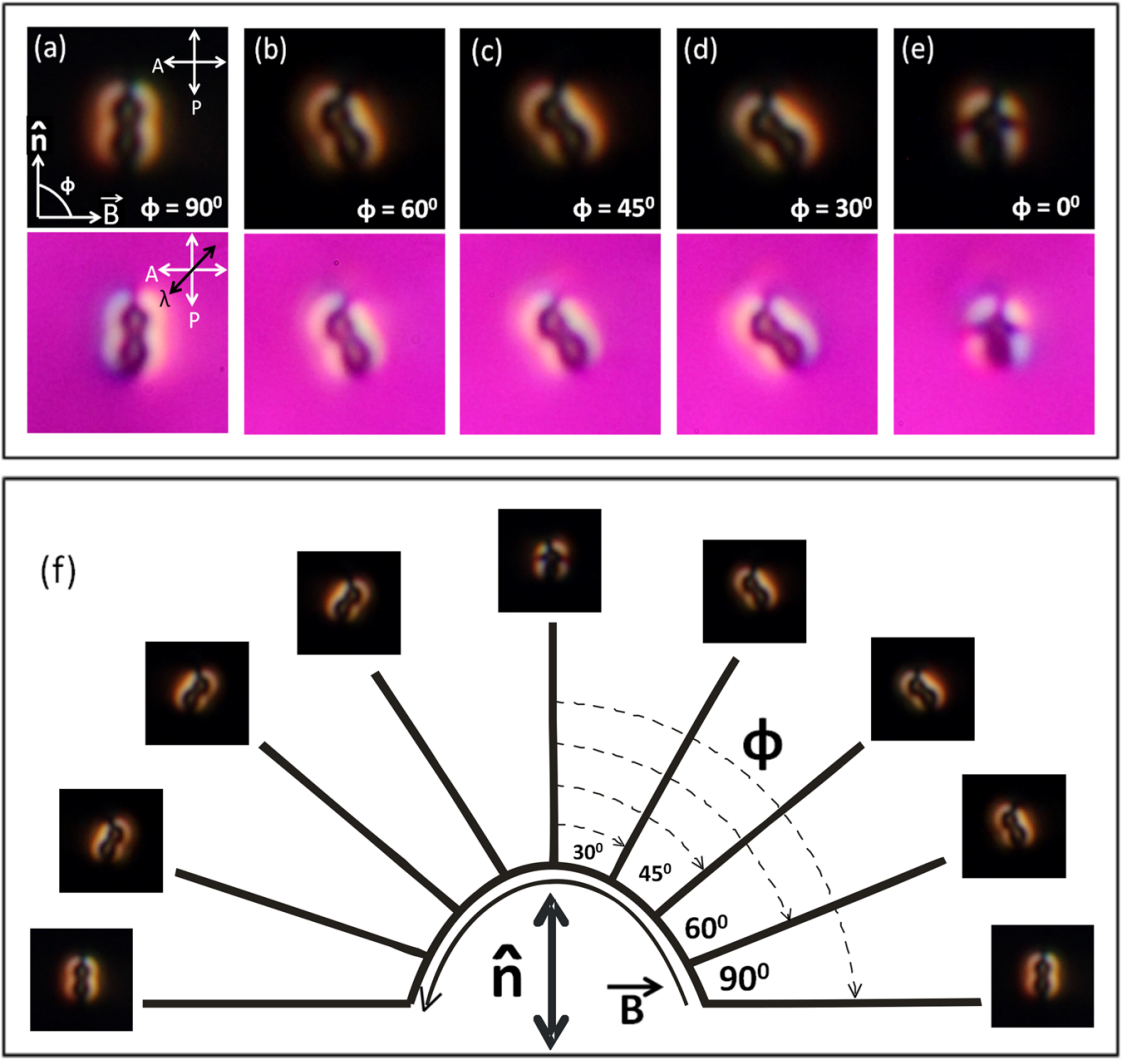
(**a**–**e**) Effect of magnetic field of strength 300 gauss on a peanut-shaped dipolar nematic colloid at different directions from 90^o^ to 0° with respect to the director, $\hat{n}$. *ϕ* is the angle between the director and the magnetic field ($\overrightarrow{B}$). Corresponding *λ*-plate images are shown underneath. (**f**) POM images when the magnetic field direction is changed from +90° to −90°. Double headed arrow at the centre represents the director $\hat{n}$.

We studied the effect of external magnetic field on the orientation and defects of dipolar colloids and estimated the magnetic dipole moment from the competing effects of elastic and magnetic torques. Two thick, disc-shaped magnets of diameter 20 mm were placed diametrically opposite in a circular track around the microscope objective, keeping the cell at the centre. This arrangement produces a uniform magnetic field with a fixed amplitude at the sample, which depends on the diameter of the circular track. The direction of magnetic field is changed by rotating the two magnets simultaneously in the track. The magnetic field at the centre is calibrated by a magnetometer. Figure 8(a–e) shows the effect of rotating magnetic field of strength 300 gauss on a dipolar colloid. Since the diamagnetic anisotropy of 5CB liquid crystal is very small the applied magnetic field does not have any effect on the orientation of the director. The orientation of the particle remains unaffected when the magnetic field is perpendicular to the long axis. As the magnetic field is rotated from 90° to 0°, the colloid rotates, keeping the point defect facing toward the rubbing direction. This suggests that the direction of magnetic moment is perpendicular to the length of the colloids. Figure 8(f) shows the response of the colloid when the magnetic field is rotated from +90° to −90° (right to left). Below ±30° the colloid tilts out of the observation plane. At *ϕ* = 0°, the tilt angle reaches maximum and the particle flips its direction from anti-clockwise to clock-wise.

**Figure 9:**
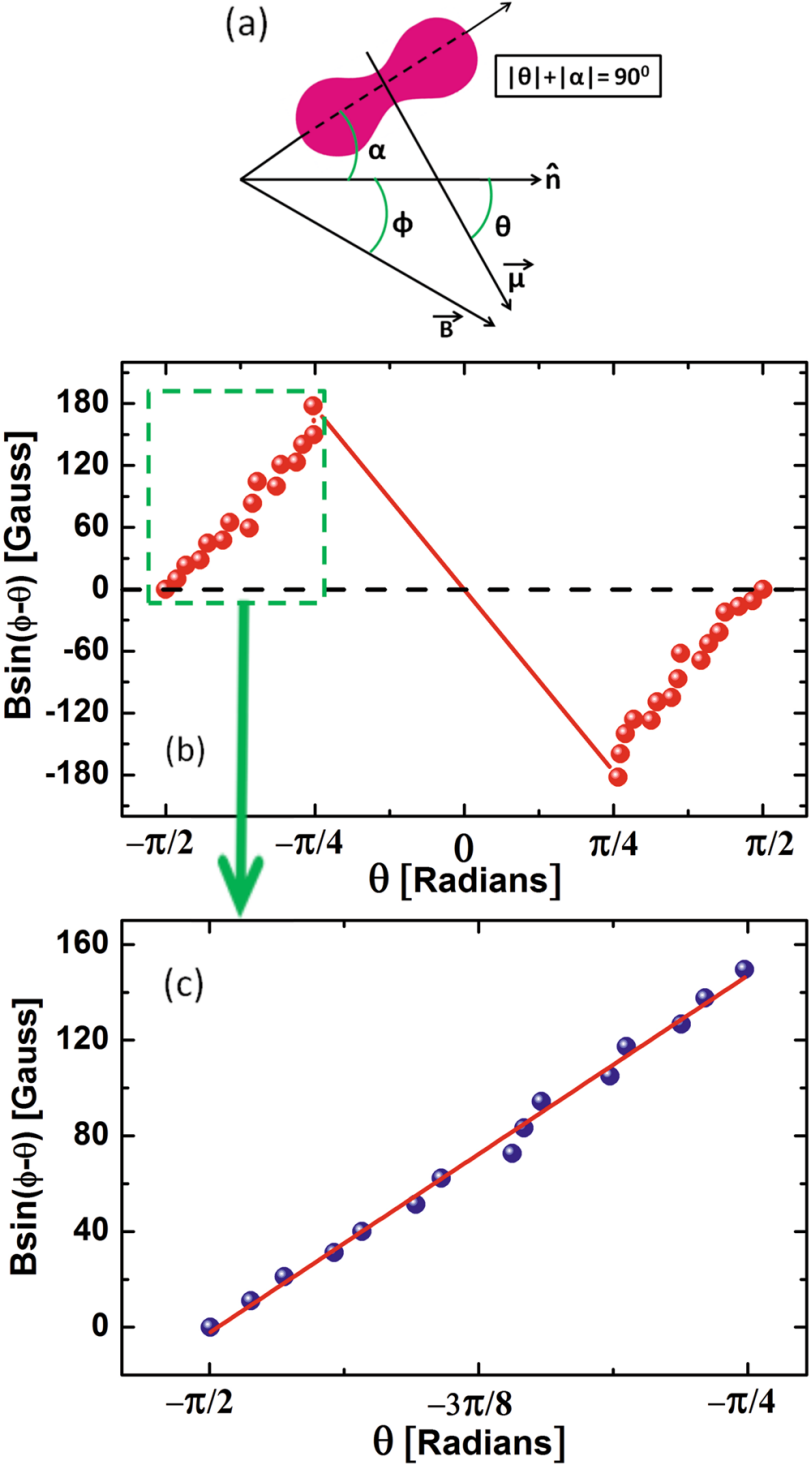
(**a**) Schematic diagram showing the direction of magnetic field and the magnetic moment with respect to the director. (**b**) Variation of magnetic torque $B\,\sin (\varphi -\theta )$ with *θ*. Measurement is restricted within ±*π*/2 to ±*π*/4 as the colloid tilts out of the plane preventing the exact measurement of *θ*. (**c**) Red line represent best linear fit to the selected experimental data as highlighted by dashed green box.

To calculate the magnetic torque on the colloid we present a simple diagram in Fig. 9(a). It shows an equilibrium orientation of the colloid when the magnetic field is applied at an angle *ϕ* with respect to the director. The long axis of the colloid makes an angle *α* with respect to the director. The angle of the magnetic moment with respect to the director is *θ*. At equilibrium the magnetic toque *μB* sin(*ϕ* − *θ*) is balanced with the gradient of elastic energy $-(\partial U/\partial \theta )$ and the torque balance equation is written as^32^:

1 $$-\partial U/\partial \theta +\mu B\,\sin (\varphi -\theta )=0$$

According to Brochard and de Gennes the elastic energy of an elongated particle in liquid crystals can be written in analogy with the electrostatic energy at a fixed potential and is given by $U=2\pi CK{\theta }^{2}$, where *K* is average Frank elastic constant and $C=L/2\,\mathrm{log}(L/d)$ is the capacitance of the particle of length *L* and diameter *d*^29^. Thus, from Eq. (1) we get, $\mu B\,\sin (\varphi -\theta )=4\pi CK\theta$. The variation of $B\,\sin (\varphi -\theta )$ with *θ* is shown in Fig. 9(b). The magnetic torque varies linearly but in a periodic manner and at *θ* = 0°, it changes direction. The slope of the best fit is given by $4\pi CK/\mu =190$  gauss/radians (Fig. 9(c)). Taking $K\simeq 5\times {10}^{-7}\,{\rm{d}}{\rm{y}}{\rm{n}}$ and $C\simeq 5\times {10}^{-4}\,{\rm{cm}}$, the estimated magnetic dipole moment $\mu \simeq 1.7\times {10}^{-11}$ emu. This is very close to the value reported by Lee *et al*. from an independent measurement of bulk sample^44^.

**Figure 10:**
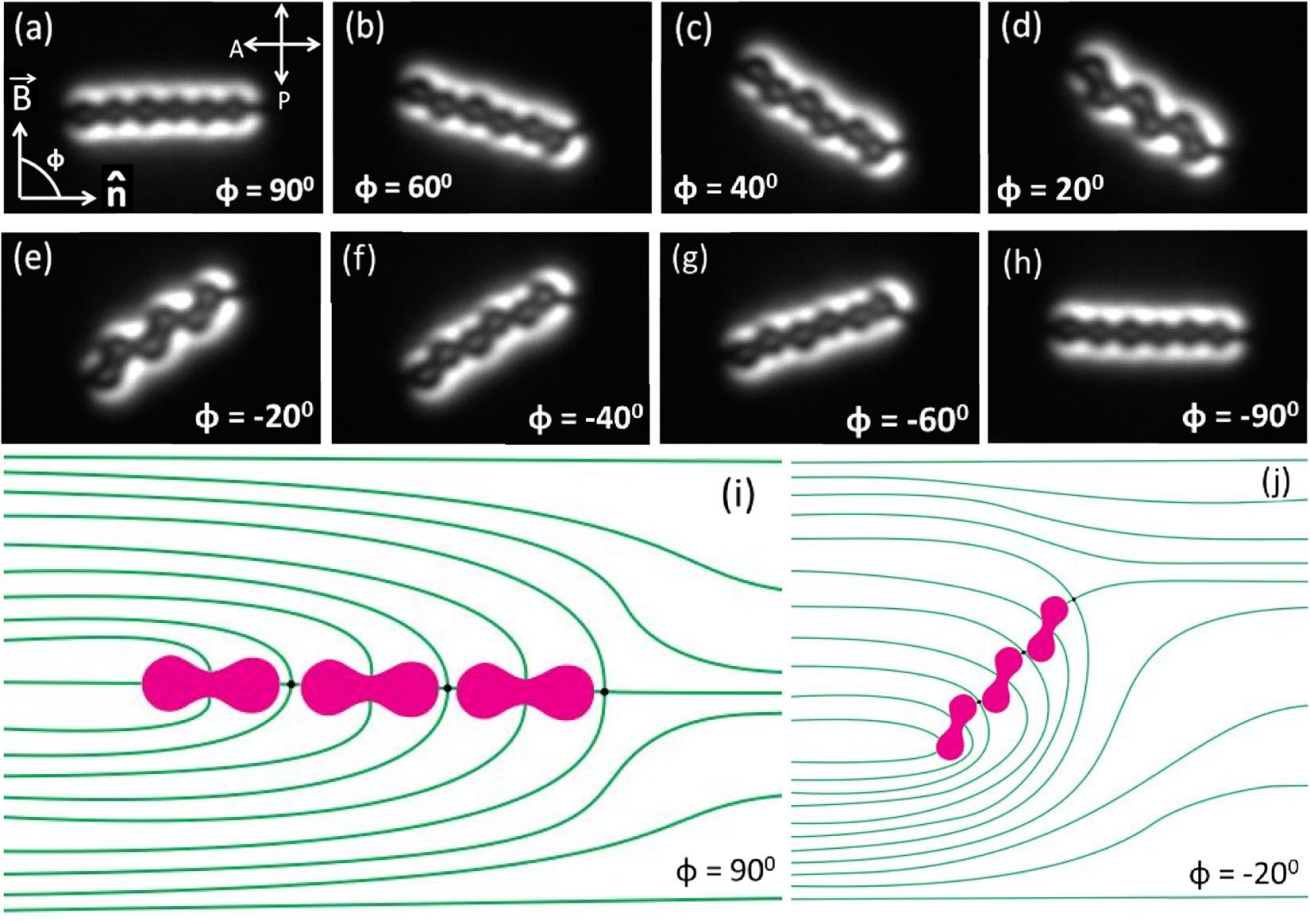
(**a**–**h**) Polarising optical microscope images showing the effect of rotating magnetic field on a dipolar chain of peanut-shaped particles. *ϕ* is the angle between the director $\hat{n}$ and magnetic field $\overrightarrow{B}$. (**i**) Schematic diagram of the director structure when the magnetic field is perpendicular to the chain as shown in texture (**a**). (**j**) Schematic diagram of the director structure when the magnetic field is rotated at the angle *ϕ* = −20°.

We also studied the effect of magnetic field on a self-assembled dipolar chain structure. Figure 10(a–h) shows a few textures of a dipolar chain, when the magnetic field is rotated around the director. The angle between the director, $\hat{n}$ and magnetic field, $\overrightarrow{B}$ is *ϕ*. The chain remains straight when the magnetic field $\overrightarrow{B}$ is perpendicular to the length of the chain, i.e., *ϕ* = 90°. The chain as a whole, tends to reorient and the colloids slide over each other as *ϕ* is decreased from 90°. The sliding is associated with a slight displacement of the point defects from their initial positions. Consequently the length becomes shorter and finally the chain flips its direction at *ϕ* = 0°. Director structures for two orientations are schematically shown in Fig. 10(i,j) respectively.

## Conclusions

In summary, peanut-shaped hematite microparticles spontaneously orient at various angles with respect to the nematic director exhibiting both dipolar and quadrupolar symmetry of elastic distortion. Among the quadrupolar colloids majority of them are tilted with respect to the plane of the cell, which has been corroborated by the computer simulation results obtained from Landau-de Gennes Q-tensor modelling. The elastic interactions are highly anisotropic and short-ranged compared to that of a pair of spherical colloids. The dipolar and quadrupolar linear chains are combined with the help of a laser tweezer to form a stable structure retaining their orthogonal orientations. The colloids have transverse magnetic dipole moments and hence their orientations are manipulated in the liquid crystal by the applied external magnetic field. The estimated magnetic moment obtained from the competing effects of elastic and magnetic torques is about 1.7 × 10^−11^ emu. Magnetic colloids with anisotropic shape have a potential use for making magnetic field responsive complex self-assembling colloids, as demonstrated on magnetically rearranging linear chain of particles. Peanut shape is structurally very similar to a pair of spherical particles, bound at a fixed distance, so the particles could also be used in a mixture of spherical and peanut-shaped particles to functionalize the particle assembly with magnetic response.

## Experimental Methods

The peanut-shaped hematite particles (*α*-Fe_2_O_3_) were synthesised based on the previously established gel-sol method^45^. They can be produced in large quantity and they are well characterised^41,42,44,45^. The particles were dispersed in 4-cyano-4′ pentylbiphenyl (5CB) liquid crystal without any surface treatment for the anchoring of liquid crystal molecules. Cells with spacing in the range of 8–10 μm were fabricated using glass plates, spin coated with polyimide, AL-1254 and cured at 180^o^. The glass plates were rubbed along a particular direction and then fixed using a UV curable optical adhesive mixed with the desired spacer. Prepared cells provide planar orientation of liquid crystal director along the rubbing direction. The LC-colloid mixture was introduced into the cells by capillary action and the textures were observed using an inverted polarising optical microscope. A laser tweezer operating at 1064 nm, is built on the same inverted microscope (Nikon eclipse Ti-U). An acousto-optic deflector was used for optical trap and hence colloid manipulation. The motion of the particle was video recorded using a CCD camera (Pixelink PLB 741F) and an appropriate software was used to track the time dependent positions of the particles^46–48^. All the experiments were performed at a temperature of 30 °C.

## Numerical Simulation

Numerical simulations were done with a finite difference scheme based on a Landau-de Gennes Q-tensor free energy density model with equal elastic constants in the form^7^:

2 $$f=\frac{L}{2}{Q}_{ij,k}{Q}_{ij,k}+\frac{A}{2}{Q}_{ij}{Q}_{ij}+\frac{B}{3}{Q}_{ij}{Q}_{jk}{Q}_{ki}+\frac{C}{4}{({Q}_{ij}{Q}_{ij})}^{2}$$

and surface free energy term ${f}_{S}=\frac{1}{2}W{({Q}_{ij}-{Q}_{ij}^{0})}^{2}$ with ${Q}_{ij}^{0}$ being the preferred *Q*-tensor consistent with the director pointing along the particle surface normal. The elastic constants are set to the well-tested set for simulating 5CB liquid crystals: *L* = 8 × 10^−12^ J, *A* = −0.172 MJ/m^3^, *B* = −2.12 MJ/m^3^, *C* = 1.73 MJ/m^3^, and relatively weak anchoring *W* = 1.0 × 10^−3^ J/m^2^. The simulations were done for particle dimensions 1.0 × 0.5 × 0.5 *μm* with computational grid resolution of 10 nm in a 300 × 300 × 300 simulation box. The particle size is smaller than in experimental setting, to make the computation feasible. However, as long as the defects are in the same positions as in the experiments, the director field is scale-independent, making the simulations suitable for reproducing the optical micrographs. The simulations of the crossed polarizer micrograph with an inserted retarder plate were performed using the Jones matrix formalism.
